# From crops to clinic: the impact of dual azole use on antifungal resistance in *Candida* and *Candida* associated yeasts

**DOI:** 10.3389/fmicb.2025.1656723

**Published:** 2025-10-03

**Authors:** Jessica C. Allison, Edel M. Hyland

**Affiliations:** School of Biological Science, Queen’s University Belfast, Belfast, United Kingdom

**Keywords:** cross-resistance, *Candida*, antifungal resistance, azoles, one health

## Abstract

Invasive fungal infections caused by pathogenic yeasts are an escalating global health crisis that demands urgent attention within a One Health framework. This review critically examines mounting evidence that widespread agricultural azole fungicide use is a key driver accelerating antifungal resistance in pathogenic yeast. We dissect the shared molecular targets and resistance pathways that underpin dangerous cross-resistance between environmental fungicides and clinical azoles. Traditionally viewed as human commensals, we provide a comprehensive account of the evidenced environmental reservoirs of yeast pathogens, including agricultural soils, wastewater, and the food chain. Ecosystems burdened by persistent azole contamination that create hotspots for resistance evolution and amplification. With antifungal treatment options rapidly diminishing and resistant infections causing rising morbidity and mortality worldwide, we identify vulnerabilities in our shared environment and consider integrated surveillance, stewardship, and environmental interventions to help preserve the efficacy of life-saving antifungals and mitigate the growing threat of fungal disease.

## 1 Current uses and limitations of antifungal drugs

Over the past several years, opportunistic fungal pathogens have become a significant threat to human health. The most prevalent invasive fungal infections are the result of *Candida* species (70%), *Aspergillus* species (10%), and *Cryptococcus* species (20%) (Fang et al., 2023). It is estimated that invasive fungal infections (IFIs) result in 3.8 million deaths globally each year (Denning, 2024), with more than 90% of deaths associated with *Candida* and *Aspergillus* species (Shafiei et al., 2020). The severity of the fungal disease is dependent on the host immune system (Shah et al., 2024), with immunocompromised individuals significantly at risk.

Agricultural environments are also highly susceptible to fungal infections, posing a serious global food security risk. It is estimated that crop fungal infections lead to 10–23% loss pre-harvest and an additional 10–20% loss post-harvest despite fungicide treatment (Steinberg and Gurr, 2020), equating to the amount of food which could sufficiently feed ~4,000 million people for a year (Olita et al., 2024). Some of the most prevalent fungal phytopathogens are associated with *Fusarium* and *Zymoseptoria* species, with *Fusarium* resulting in a global crop yield loss of ~80% (Amar, 2021), and *Z. tritici* causing up to 50% yield losses of wheat annually (Stukenbrock and Gurr, 2023).

Our arsenal of drugs to combat fungal disease is very limited. There are four primary antifungal drug classes used to treat IFIs in the clinic: azoles, polyenes, pyrimidine analogs (5-FC), and echinocandins (Souza et al., 2025). In agricultural settings, the situation is better, with fungicide classes being divided according to 52 different modes of action (Lamberth, 2022). The five major classes of fungicides include benzimidazoles, demethylation inhibitors (DMIs), quinone outside inhibitors (Qols), quinone inside inhibitors (Qils) and succinate dehydrogenase inhibitors (SDHIs) (Corkley et al., 2022).

### 1.1 Mechanism of action of ergosterol biosynthetic inhibitors

Azole antifungals represent one of the most widely used drug classes in clinical medicine, targeting the ergosterol biosynthesis pathway (Monk et al., 2020). Ergosterol is the prevalent sterol in fungal cell membranes, with crucial functions in the regulation of membrane structure, fluidity and permeability (reviewed in Rodrigues, 2018). Azoles alter membrane function by inhibiting the cytochrome P450-dependent enzyme, 14-⍺-demethylase, encoded by *ERG11* in yeast and *CYP51* in moulds (Cowen et al., 2014). Azole antifungals are mostly fungistatic towards yeasts, arresting their growth as opposed to killing them, whereas they can be fungicidal for some moulds (Geissel et al., 2018). However, under certain conditions azoles are fungicidal against yeast. For example, in *C. albicans,* itraconazole was shown to trigger apoptosis (Lee and Lee, 2018).

Despite their fungistatic nature, azoles remain the first line of therapy in clinical settings due to their low incidence of toxicity compared to other drug classes (Osa et al., 2020). In agricultural settings, the comparable fungicide drug class are demethylase inhibitors, or DMIs (Stenzel and Vors, 2019), as they are largely comprised of triazole and imidazole compounds (FRAC, 2025) and also target the enzyme 14-⍺-demethylase, encoded by CYP51 in phytopathogens (Mair et al., 2020). DMIs are the most widely used fungicide in agriculture as they exhibit a broad range of activity with high efficacy levels (Wang et al., 2024). For this review we will use the broad term azole to refer to both clinical antifungals and agro-chemical fungicides that target ergosterol biosynthesis through CYP51/ERG11 inhibition.

### 1.2 Structural conservation of azole targets - implications for cross-reactivity

**Figure 1:**
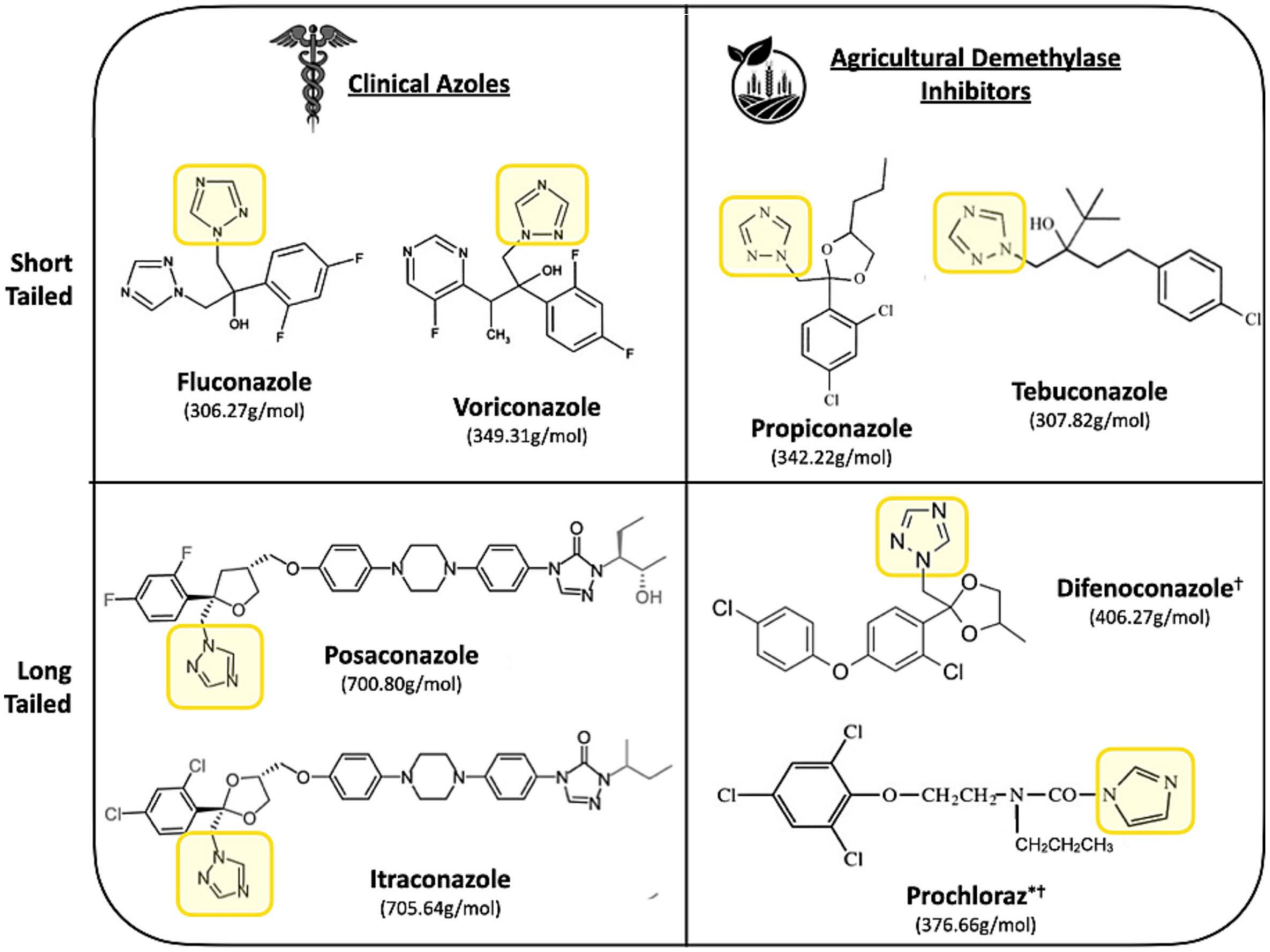
Chemical structures of short-tailed (fluconazole, voriconazole, propiconazole, and tebuconazole), and long-tailed (itraconazole, posaconazole, difenoconazole, and prochloraz) ergosterol biosynthesis inhibitors. The common structure is highlighted by a yellow box, featuring a nitrogen-containing ring with two adjacent CH or N ring members. *imidazole-based DMIs, ^†^fungicides which are classified as long-tailed based on potency, not length.

As depicted in Figure 1, both clinical and agricultural azoles share similar structures (Monk et al., 2020). A key structural feature of all azoles is a nitrogen ring with two adjacent CH or N ring members which enables non-competitive binding to the iron-heme active site of the ERG11/CYP51 enzyme (Yoshida and Aoyama, 1987; Podust et al., 2001). Extensive studies on the molecular interactions reveal that hydrophobic forces between the drug and the non-polar residues of the enzyme’s active site primarily drive this binding (Reviewed in Ruge et al., 2005). Azoles can be classified as short-tailed or long-tailed, based on both the length of the compound but also the extent to which they bind to their target. Long-tailed azoles forming additional hydrophobic interactions along the active site, leading to increased binding affinities and drug potency (Sagatova et al., 2016; Warrilow et al., 2010). This distinction is clear for clinical azoles, however most DMIs are considered short-tailed (Gisi, 2022). Although not explicitly classed as long-tailed azoles, some DMIs (difenoconazole and prochloraz) can still demonstrate long-tailed drug potency (Zhang et al., 2021; Strickland et al., 2022).

Despite major differences in their targeted fungus, both clinical and agricultural azoles can cross-react due to the high structural conservation of cytochrome P450 enzymes in biology (Nelson, 2018; Srejber et al., 2018; Lepesheva and Waterman, 2011). Indeed, 42% of amino acid sites are invariant among fungal P450 homologues, many of which occupy structurally critical positions (Lepesheva et al., 2010; Zhang et al., 2019). Furthermore, residues involved in the azole-enzyme interaction are evolutionarily conserved between ERG11 and CYP51 homologues. For example, an active site tyrosine that forms critical H-bonds with azoles are conserved between *Candida albicans ERG11* (Y132) and the *CYP51* homologues of the phytopathogens, *Z. tritici* (Y137), *Penicillium digitatum* (Y126) and *Blumeria graminis* (Y136). Additionally, there is a core set of invariant non-polar residues on the azole binding surface of many ERG11/CYP51 homologues (Sagatova et al., 2016; Parker et al., 2014; Shi et al., 2020; Li et al., 2011). Given such structurally similarities, it is unsurprising that both clinical azoles and agricultural fungicides bind to and inhibit *C. albicans* Erg11p to similar extents, with comparable Kd and IC_50_ values, respectively, (Parker et al., 2014). Theoretically, this means that DMI exposure can exert strong selective pressure on *C. albicans*, and potentially other pathogenic yeasts. Encouraging them to adapt to azole. This raises significant concerns, as widespread agricultural use of DMIs may contribute to azole resistance in environmental pathogenic yeast populations, potentially compromising clinical treatment options for candidiasis (Gomez Londono and Brewer, 2023).

**Figure 2:**
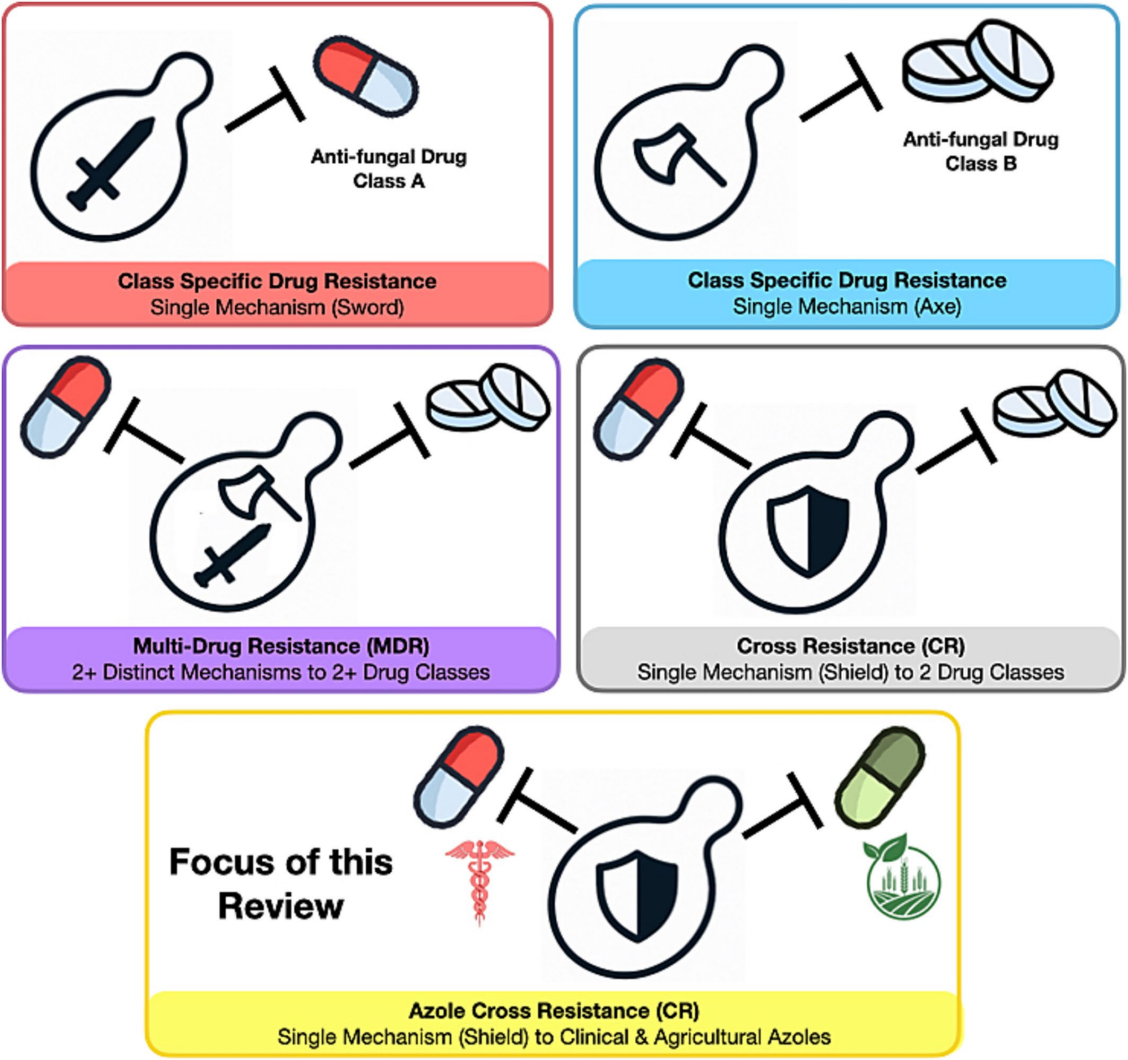
Resistance profiles in fungal pathogens. The image illustrates the potential resistance profiles of pathogenic yeast to antifungal drugs.

This review focuses on the impact of DMIs on antifungal drug resistance in pathogenic yeast. Specifically, we are only considering yeast from the Ascomycota phylum, with a focus on *Candida albicans*, *Candida parapsilosis*, *Candida tropicalis*, and *Candida* associated yeasts *Pichia kudriavzevii* (*Candida krusei*), *Candidozyma auris* (*Candida auris*), Meyerozyma *guilliermondii* (*Candida guillermondii*), and *Nakaseomyces glabratus* (*Candida glabrata*), these will be referred to collectively as pathogenic yeasts. We will use ‘cross-resistance’ to mean dual resistance towards azole antifungals and fungicides based on a single mechanism, and ‘multi-drug resistance’ as the ability of a single species to be resistant to multiple classes of antifungals *or* fungicides (Figure 2). Our aim is to address the following questions related to azole cross-resistance in *Candida* species.

1. Do *Candida* species exhibit shared resistance mechanisms to clinical and agricultural azoles that enable cross-resistance?
2. Are environmental reservoirs of *Candida* spp. exposed to agricultural DMIs?
3. Are there plausible transmission routes through which humans are exposed to DMI-adapted *Candida* strains*?*

By addressing these questions, based on our current knowledge, we aim to critically evaluate the threat posed by agricultural DMI use on clinical azole resistance and the epidemiology of fungal infections.

## 2 Mechanisms of resistance to azoles in pathogenic yeast

Acquired fungal drug resistance arises from the selection of genetic adaptive mutations by drug exposure (Czajka et al., 2023). In the context of ergosterol biosynthesis inhibitors, acquired resistance in pathogenic yeast species typically evolves via either target-site (direct) or non-target-site (indirect) mechanisms. Most clinically relevant fungal species exhibit both routes, although some show a bias toward one, potentially due to a factors such as species-specific mutational bias (reviewed in Osset-Trénor et al., 2023). Direct resistance mechanisms to azoles commonly involve point mutations within *ERG11*/*CYP51*, the genes encoding the target enzyme, that reduce drug binding affinity. Overexpression of *ERG11* is another direct mechanism, effectively increasing the amount of target enzyme and necessitating higher drug concentrations to achieve inhibition (Zavrel et al., 2017; Handelman et al., 2021). Indirect resistance mechanisms primarily entail enhanced drug efflux, mediated by upregulated expression of transmembrane transporters. These often result from gain-of-function mutations in either the promoter regions of efflux pump genes or in transcription factors that regulate their expression (Hokken et al., 2019). While these mechanisms represent the most well-characterized routes to azole resistance, they do not encompass the full complexity of resistance evolution in pathogenic yeast. For example, other studies have demonstrated that changes in ploidy (Ahmad et al., 2014), and the roles of DNA mismatch repair pathways (Legrand et al., 2007) can also alter azole susceptibility. Several comprehensive reviews have catalogued the diversity of mutations and regulatory changes contributing to this phenotype (Bhattacharya et al., 2020; Lee et al., 2021; Chaabane et al., 2019; Fisher et al., 2022).

### 2.1 Experimentally induced azole cross resistance in pathogenic yeast

Despite the pressing need to understand molecular cross-resistance mechanisms in yeast pathogens, evidence remains limited. Most studies have employed *in vitro* induction assays: Yeast isolates are first exposed to agricultural DMIs, then tested for azole susceptibility. Faria-Ramos et al. (2014) demonstrated that a 90-day exposure to the DMI prochloraz led to stable cross-resistance to fluconazole and posaconazole—but only in *Nakaseomyces. glabratus* isolates (Faria-Ramos et al., 2014). Similarly, Rocha et al. (2016) demonstrated that resistance to fluconazole, itraconazole and voriconazole evolves in *Candida parapsilosis* following 49-day exposure to the DMI tetraconazole (Rocha et al., 2016). Such findings were expanded by the same group in 2019 showing that only 15-day exposure to the DMIs tebuconazole and tetraconazole was sufficient to cause fluconazole resistance in *C. orthopsilosis*, *C. metapsilopsis* and *C. parapsilosis* (Brilhante et al., 2019). Together, these findings robustly support that exposure to DMIs can select for mutations that confer resistance to clinical azoles in multiple *Candida* species under experimental conditions.

Interestingly, exposure to DMIs does not universally result in cross-resistance in yeast. For instance, although prochloraz exposure induced resistance to DMIs in *C. albicans* and *C. parapsilosis*, no corresponding decrease in susceptibility to fluconazole or posaconazole was observed, indicating an absence of clinically relevant cross resistance in these strains (Faria-Ramos et al., 2014). Similarly, prolonged growth in the presence of tebuconazole or tetraconazole did not confer resistance to itraconazole or voriconazole in *C. parapsilosis* or *C. orthopsilosis* (Brilhante et al., 2019). These findings suggest that the development of cross resistance following DMI exposure is not uniform across yeast species or azole compounds and may be contingent upon species/strain-specific responses or compound-specific effects. The difference in chemical structure of azole compounds may also impact cross-resistance patterns. For example, mutations which select for resistance towards short-tailed triazoles will not confer resistance towards long-tailed azoles (Toepfer et al., 2023; Rallos and Baudoin, 2016). It is also worth noting that such *in vitro* assays are inherently limited by the stochastic nature of mutational events that drive resistance evolution (Hawkins et al., 2016), underscoring the need for broader and more systematic investigations to clarify the conditions under which cross resistance may emerge.

### 2.2 Molecular basis of azole cross-resistance in yeast pathogens

Gene expression analyses of experimentally derived cross resistant yeast strains have begun to elucidate the molecular basis of this phenotype. In *Nakaseomyces glabratus*, exposure to prochloraz induces overexpression of the ATP-binding cassette (ABC) transporters *ngPDH1* and *ngCDR1*, as well as the transcription factor *ngPDR1*, which regulates their expression (Faria-Ramos et al., 2014). These mechanisms mirror those commonly associated with resistance to clinical azoles. Notably, this study also identified overexpression of *ngYOR1*, encoding an ABC transporter not previously linked to azole resistance, suggesting the existence of a potentially unique cross resistance pathway in *N. glabratus* (Faria-Ramos et al., 2014). In *Candida parapsilosis*, however, the molecular responses to DMI exposure appear more variable. One study reported increased expression and activity of the efflux transporter *cpCDR1* following tetraconazole exposure (Rocha et al., 2016). Conversely, a separate investigation found that *cpCDR1* was downregulated in the *C. parapsilosis* species complex after exposure to tebuconazole and tetraconazole, while *cpERG11* expression was upregulated (Brilhante et al., 2019). Despite these differences, both studies implicate efflux transporters in the cross resistance response of *C. parapsilosis* and *C. metapsilosis*, suggesting a potential bias toward indirect resistance mechanisms in these closely related species (Rocha et al., 2016; Brilhante et al., 2019). Collectively, these findings support the view that DMI exposure can dysregulate indirect resistance pathways, primarily involving efflux transporters and their regulators (summarized in Table 1).

**Table 1:** Summary of studies detecting cross-resistance in *Candida* species.

Species	Country/ Sample type	Initial azole exposure	Tested azole sensitivity	Selected Pressure	Mechanism of Cross-Resistance	References
*Candida parapsilosis*	Brazil, Type Strain	Tetraconazole	Fluconazole	Experimental Defined	Upregulation of efflux pumps through increased activity of *CDR* transporters.	Rocha et al. (2016)
*Candida parapsilosis* Complex	Brazil, Type strain, FLU^SEN-^sensitive clinical isolates	Tetraconazole, Tebuconazole	Fluconazole, Itraconazole, Voriconazole	Experimental Defined	Overexpression of *ERG11*, downregulation of *CDR1*, and overexpression of *MDR1*.	Brilhante et al. (2019)
*Nakaseomyces glabratus*	Portugal, Clinical Isolates	Prochloraz	Fluconazole, Posaconazole	Experimental Defined	Upregulation of ABC transporters *PDH1*, *YOR1*, *CDR1*, and *SNQ2*, and a G727A point mutation in *PDR1* transporter gene.	Faria-Ramos et al. (2014)
*Candida albicans*	Germany, Clinical and veterinary Isolates		Fluconazole, Ketoconazole, Itraconazole, Voriconazole Tebuconazole, Fluquinconazole, Triadimenol, Penconazole	Environmentally Defined	ns	Muller et al. (2007)
*Candida tropicalis*	Taiwan, Fruit isolates	Triadimenol	Fluconazole	Environmentally	ns	Lo et al. (2017)
*Candida tropicalis*	Taiwan, Clinical and Soil isolates	Tebuconazole, Fluquinconazole, Triadimenol, Penconazole	Fluconazole	Environmentally Defined	ns	Yang et al. (2012)
*Candida albicans*	Poland, Type Strains	Epoxiconazole, Fenpropimorph	ns	Experimental Defined	Genetic instability leading to phenotypic heterogeneity. Cross resistance not tested explicitly.	Potocki et al. (2020)

Experimentally defined reflects azole resistance induction assays, whereas environmentally defined reflects natural azole exposure. DMIs are in blue, ns, not specified.

Exposure to DMIs also elicits broader physiological and genomic responses in yeast species. Potocki et al. (2020) reported that treatment with the fungicide epoxiconazole led to increased ROS, altered fatty acid and phospholipid composition, reduced biofilm formation, and heightened DNA damage across four species—*C. albicans*, *N. glabratus*, *C. tropicalis*, and *C. pulcherrima*—resulting in genetically and phenotypically heterogeneous populations (Potocki et al., 2020). Notably, diminished biofilm formation was also observed in tebuconazole-treated *C. parapsilosis* (Rocha et al., 2016). In addition, Hu et al. (2025) demonstrated that *C. tropicalis* exposed to tebuconazole resulted in cross-resistance to both fluconazole and voriconazole. Furthermore, these resistance strains displayed genomic instability manifesting as ploidy variation—including haploidization, and a reduction in growth rate (Hu et al., 2025). Such genomic plasticity underscores a potential mechanism by which environmental DMIs contribute to the evolution of clinically relevant azole resistance (Hu et al., 2025).

## 3 Environmental reservoirs of yeast species

For fungicide-exposed human pathogens to drive clinical resistance, two conditions must be met. (1) Human fungal pathogens must actively grow in agricultural environments where they are exposed to fungicides, and (2) Humans must routinely come into contact with these environmentally derived strains. Both conditions are met in the case of *Aspergillus* species (Rhodes et al., 2022). These opportunistic pathogens are considered ubiquitous in the environment and have been detected in soil, organic matter, air, and water (Paulussen et al., 2017). *Aspergillus* spp. produce airborne spores which are easily inhaled by humans. Indeed, it is estimated that humans inhale 500–100,000 pathogenic spores daily (Salazar-Hamm et al., 2022), and not surprisingly, inhalation is the primary route of infection for invasive aspergillosis (Park et al., 2019). This means that *Aspergillus* spores from environments regularly exposed to DMIs, offer a direct pathway for potentially resistant fungal pathogens to move from the environment to humans.

For opportunistic yeast species the role of fungicide exposure in driving cross-resistance is less obvious. Firstly yeast species are not widespread in the environment (Kumamoto et al., 2020), and are not classed as significant environmental pathogen (Ratnadass and Martin, 2022) suggesting limited exposure to DMIs. Opportunistic yeast pathogens, such as *Candida* spp. are primarily commensal organisms existing within the natural microflora of the gastrointestinal tract, skin, and oral and vaginal mucosa of humans (Boonsilp et al., 2021; Limon et al., 2017). Similarly, in animals, opportunistic yeasts can be part of their microbiome. *Candida tropicalis* has been isolated from the microbiome of healthy ruminants (goats, sheep), horses, shrimp, sirenians (manatees), and dwarf sperm whales (Cordeiro Rde et al., 2015). Additionally *C. albicans* and non-*albicans* strains considered commensals have been isolated from avian species; Galliformes, Anseriformes, Columbiformes, and Passeriformes (Salazar-Hamm et al., 2025; Talazadeh et al., 2022; Talazadeh et al., 2023), psittacine, and rheas (Cordeiro Rde et al., 2015).

This commensal lifestyle however, does mean that many opportunistic yeasts can be environmental contaminants, due to shedding from their host and animal excretions (Carpouron et al., 2022; Branda Dos Reis et al., 2024; Rosario Medina et al., 2017). Although this fungal contamination does not indicate a true environmental niche, the main point is that regardless of their origin, clinically relevant yeast species have been isolated from non-clinical environmental reservoirs such as soil, water, fruit, trees, and plants (Akinbobola et al., 2023), as summarized in Table 2. This suggests that environmental exposure to DMIs, while likely a lot lower than for *Aspergillus*, cannot be ruled out entirely.

**Table 2:** Summary of *Candida* species isolated from environmental samples.

Species	Soil/Sand			Aquatic			Plants and Fruit			Animals			References
	Agricultural Soil	Urban Topsoil	Sand	Fresh Water	Sea Water	Waste Water	Trees	Plant Debris	Fruit	Domestic Livestock	Domestic Pets	Birds	
*Candidozyma auris*	●		●	●	●	●			●		●		Akinbobola et al. (2023), Arora et al. (2021), Babler et al. (2023), Yadav and Heitman (2022), Yadav et al. (2022), Zulli et al. (2024), White et al. (2024), and Yadav et al. (2023)
*Candida albicans*	●			●	●	●	●	●	●	●	●	●	Talazadeh et al. (2022), Sautour et al. (2021), Chaieb et al. (2011), Steffen et al. (2023), Mataraci-Kara et al. (2020), Hamlin et al. (2019), Opulente et al. (2019), Yadav and Heitman (2022), Uhitil et al. (2009), Mba and Nweze (2019), Jin and Lin (2005), and Reagan et al. (2019)
*Nakaseomyces glabratus*	●			●		●	●	●	●	●	●	●	Talazadeh et al. (2022), Al-Yasiri et al. (2016), Steffen et al. (2023), Mataraci-Kara et al. (2020), Opulente et al. (2019), Carvalho et al. (2014), Gabaldón and Fairhead (2019), Drumonde-Neves et al. (2017), Mba and Nweze (2019), Yadav et al. (2023), and Reagan et al. (2019)
*Candida parapsilosis*	●	●	●		●		●	●	●	●	●	●	Glushakova et al. (2024), Kaewkrajay et al. (2021), Maganti et al. (2012), Opulente et al. (2019), Lo et al. (2017), Du et al. (2018), Mba and Nweze (2019), Jin and Lin (2005), Reagan et al. (2019), Branco et al. (2023), and Queiroz-Aaltonen et al. (2021)
*Candida tropicalis*	●		●		●	●	●	●	●		●	●	Lima et al. (2022), Chaieb et al. (2011), Zuza-Alves et al. (2016), Mataraci-Kara et al. (2020), Opulente et al. (2019), Carvalho et al. (2014), Chen et al. (2023), Yadav et al. (2023), Jin and Lin (2005), Reagan et al. (2019), and Queiroz-Aaltonen et al. (2021)
*Meyerozyma guilliermondii*						●					●	●	Talazadeh et al. (2022), Mataraci-Kara et al. (2020), Jin and Lin (2005), and Reagan et al. (2019)
*Pichia kudriavzevii*									●	●	●	●	Chen et al. (2023), Du et al. (2018), Jin and Lin (2005), Reagan et al. (2019), and Queiroz-Aaltonen et al. (2021)

### 3.1 Yeast species in soil

Soil may serve as a potential environmental reservoir for opportunistic yeast species. Research shows that yeast favour agricultural and grassland soils, likely due to their copiotrophic lifestyle – thriving on simple sugars and tolerating low oxygen conditions (Yurkov, 2018). Many yeasts also contribute to soil health by participating in nutrient cycling and transformation, organic matter decomposition and soil fertilisation (Samarasinghe et al., 2021). For instance, *Candida tropicalis* HY (CtHY), isolated from rice rhizosphere, produces plant growth regulators and is commonly used as biofertilizer (Amprayn et al., 2012).

Evidence suggests soil-dwelling yeasts are globally distributed (Lima et al., 2022; Glushakova et al., 2024)*. Candida tropicalis* has been isolated from agricultural fields, forest and sludge soil across Taiwan, China, Brazil, USA, UK and Ireland (Lima et al., 2022) and *C. parapsilosis* from urban topsoil in Moscow (Glushakova et al., 2024). While *N. glabratus* is often recovered from soil, its presence is attributed to contamination from yellow-legged gull faeces (Al-Yasiri et al., 2016). Regardless of how these yeast species reach soils, it clearly survives in this environment, *C. albicans* for example was shown to replicate in French soils for up to 30-days (Sautour et al., 2021).

### 3.2 Yeast species in aquatic environments

An increasing number of studies associate yeast pathogens with aquatic environments. The most notable example is *Candidozyma auris* (formally *Candida auris*) a recently identified species known for its thermotolerance and halotolerance. These traits have led researchers to hypothesize that, *C. auris* originated in marine or freshwater ecosystems (Akinbobola et al., 2023; Sharma and Chakrabarti, 2020; Casadevall et al., 2019). Supporting this, Arora et al. (2021), isolated *C. auris* from sandy beaches and salt marshes in India’s coastal wetlands, while Escandon (2022) reported *C. auris* presence in estuaries in Colombia. Additionally, *C. auris* shares a close relationship with *Candida haemulonii*, a species previously isolated from seawater off the coast of Portugal and the skin of dolphins (Garcia-Bustos et al., 2024). This phylogenetic link further suggests that *C. auris* originated in aquatic ecosystems. Alarmingly, these environmental *C. auris* strains often display multidrug resistance to clinical azoles, underscoring the threat they pose to human health (Arora et al., 2021). Furthermore, Akinbobola et al. (2024) observed that *C. auris* can colonise plastic pollutants in marine environments for up to 30 days, without compromising pathogenicity (Akinbobola et al., 2024).

Other opportunistic yeasts, including *C. albicans*, *C. tropicalis*, and *C. parapsilosis* also exhibit halo-tolerance, suggesting an ability to survive in marine niches (Chaieb et al., 2011; Zuza-Alves et al., 2017; Kaewkrajay et al., 2021). Accordingly, *C. albicans* persists in Tunisian seawater for up to 200 days (Chaieb et al., 2011), and *C. tropicalis* has been isolated from sand and coastal waters of Miami and Brazil (Zuza-Alves et al., 2016; Vogel et al., 2007). A startling 2024 study has uncovered a rising threat of fungal infections among cetacean marine species, including cases involving drug-resistant *Candida* yeast (Garcia-Bustos et al., 2024). Furthermore, *C. tropicalis* and *C. parapsilosis* are associated with marine sponges in the South China Sea, isolated from seawater, sea sediments, marine ecosystems, and beach sand (Kaewkrajay et al., 2021). Worryingly, fluconazole resistant *N. glabratus* has been found in river water, alongside *C. albicans,* and both are now classified as river water contaminants in South Africa (Steffen et al., 2023).

Wastewater and sewage effluent is another significant aquatic reservoir for human fungal pathogens (Assress et al., 2019). It has been suggested that the phenotypic plasticity exhibited by many yeast species such as biofilm formation and their ability to adapt rapidly to changing environments, may explain their persistence in wastewater (Buratti et al., 2022; Marta and Alina, 2017). Given their role as gut commensals, it is unsurprising that 0.1–1% of human faecal matter is fungal in origin (Qin et al., 2010), including major opportunistic yeast pathogens (Gurleen and Savio, 2016). Indeed, one study investigating *Candida* species present in wastewater from Brazil identified six *Candida* species within sewage samples (Correa-Moreira et al., 2024). Similarly, *C. albicans, N. glabratus, C. tropicalis, Meyerozyma guilliermondii,* and *C. auris* are routinely isolated from both hospital and community wastewater sources (Mataraci-Kara et al., 2020; Babler et al., 2023). Indeed *C. auris* has been found in 190 general wastewater treatment plants across 41 US states (Zulli et al., 2024).

### 3.3 Yeast species and plants

In recent years growing evidence has highlighted the association between opportunistic yeast and plants. While much of this attention has focused on the detection of human pathogens in the food chain, especially fruit, reports also document the incidence of yeast directly on trees and in plant litter (Bensasson et al., 2019; Hamlin et al., 2019; Maganti et al., 2012; Opulente et al., 2019). Research from North America has reported various species including *C. albicans* (Hamlin et al., 2019), *C. parapsilosis (*Maganti et al., *2012**)*, *C. tropicalis*, and *N. glabratus* (Carvalho et al., 2014) on trees such as oak, pine, maple, ash, cedar and birch. In some cases, these associations appear species-specific, with one study noting that *C. parapsilosis* exclusively colonized pine trees (Maganti et al., 2012). *Nakaseomyces glabratus* and *C. tropicalis* also associate with cedar and birch trees (Carvalho et al., 2014). All four species have also been isolated from decaying plant matter, including duff and leaf litter (Opulente et al., 2019).

Even more concerning is the repeated detection of yeast pathogens on raw fruit, which can serve as a direct route of human exposure. Drug-resistant strains such as fluconazole-resistant *C. auris* have been isolated from apples in India (Yadav and Heitman, 2022; Yadav et al., 2022). In Taiwan, *C. tropicalis* and *Pichia kudriavzevii* were found on a range of fruits including pears, mangoes, melons, and guavas (Chen et al., 2023). Other studies have reported *C. tropicalis* on bananas and waxed apples, *P. kudriavzevii* on tomatoes, and *C. parapsilosis* on pears (Lo et al., 2017). Though less common*, N. glabratus* has been recovered from crushed grapes and during wine fermentation (Gabaldón and Fairhead, 2019; Drumonde-Neves et al., 2017). While *C. albicans* has been isolated from a freshly harvested apple in India (Yadav and Heitman, 2022), and from fresh orange juice in Croatia (Uhitil et al., 2009).

While some argue that these findings may result from human handling, since many yeast species are part of the skin microbiome (Yadav and Heitman, 2022). This explanation does not lessen the risk. Importantly, fruit can serve as a nutritional source for yeasts permitting their growth and persistence, even those transferred from humans. Moreover, many fruits especially non-organic ones, are treated with post-harvest fungicides to prevent spoilage (Yadav et al., 2022). This creates an environment where opportunistic yeast pathogens, regardless of how it arrived on the surface, are exposed to drugs that can select for resistance, possibly driving cross resistance (Crump and Edlinds, 2004).

These observations challenge the conventional view of yeast species as obligate human commensals and underscore the necessity of re-evaluating their ecological breadth (Sautour et al., 2021; Sharma and Chakrabarti, 2020). Although methodological limitations may obscure the true prevalence of *Candida* and other yeast species in environmental samples, the diversity of niches yielding positive detections is striking and emphasizes the importance of further research into their environmental biology. It remains unclear whether these fungi represent long-term components of these ecosystems or are recent introductions via anthropogenic or zoonotic shedding. Both scenarios are likely contributory. Notably, recent genomic evidence suggests that environmental *C. albicans* isolates recovered from oak trees exhibit significant genetic divergence from clinical strains, implying independent evolutionary trajectories (Salazar-Hamm et al., 2025). Despite the frequent recovery of pathogenic yeast species from non-clinical reservoirs, the implications of their environmental persistence for the epidemiology of candidiasis remain poorly understood (Czajka et al., 2023).

## 4 A one health perspective on antifungal resistance

The One Health framework emphasizes the interconnectedness of human, animal, and environmental health, recognizing that the health of each domain is inextricably linked (Mackenzie and Jeggo, 2019). In the context of antifungal resistance in opportunistic yeast, a One Health approach provides a critical lens through which to examine the relationship between the agricultural use of DMI and the declining efficacy of clinical azoles in treating candidiasis. Here, we define this perspective as encompassing the environmental evolution and potential transmission of azole cross-resistant yeast pathogens to humans.

**Figure 3:**
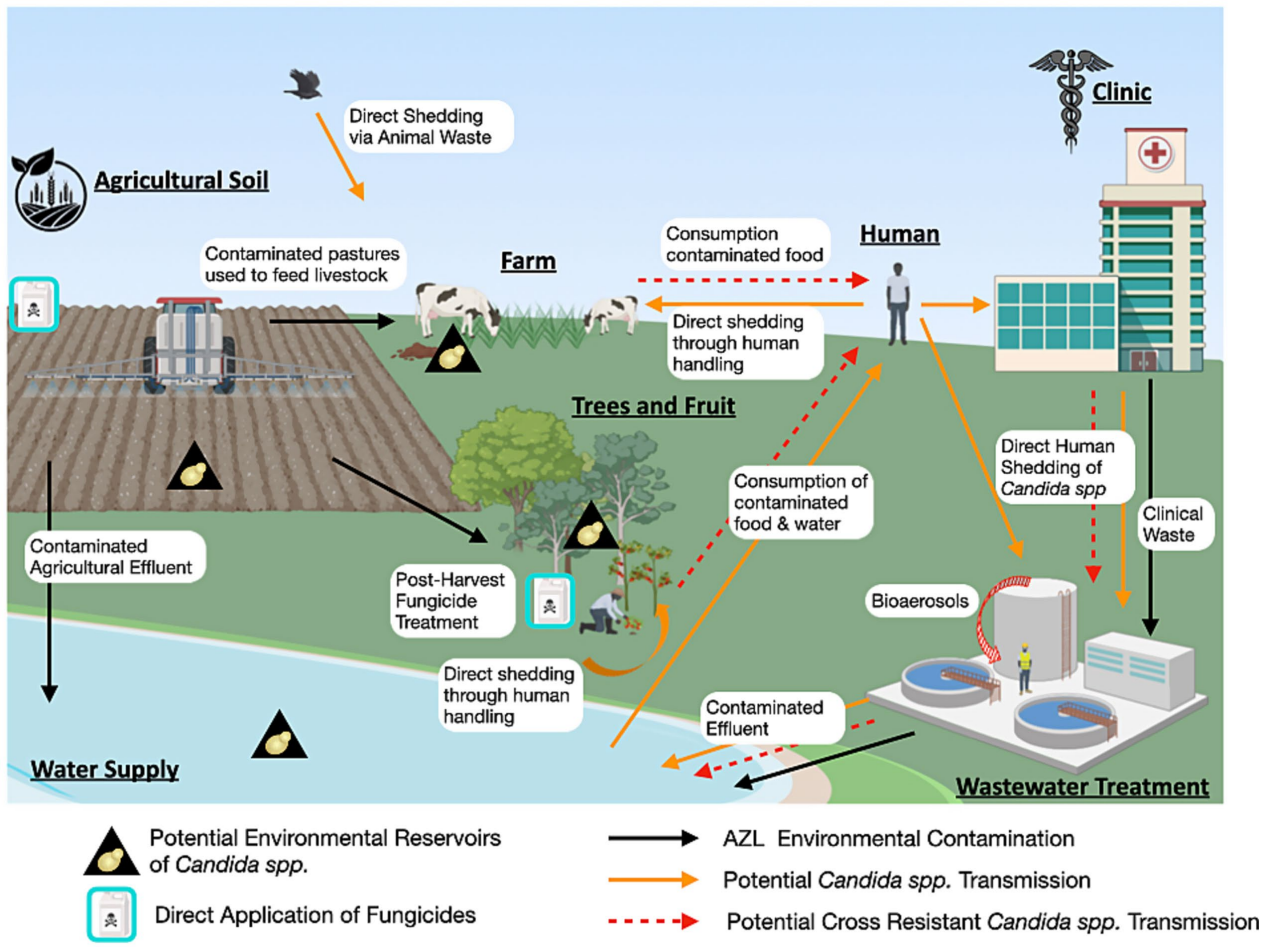
One Health perspective of *Candida* azole resistance. This diagram illustrates the non-clinical reservoirs of *Candida* species, the environmental contamination of clinical azoles and DMIs, as well as the potential routes of transmission of cross-resistant strains (Figure generated using Biorender.com).

If yeast pathogens are capable of actively growing in non-host environments, and DMI exposure can select for mutations that confer cross-resistance, then the emergence of environmentally derived resistant strains in human populations becomes a plausible scenario. The detection of azole-resistant *Candida* isolates in treatment-naïve patients further supports the notion that environmental acquisition of resistance may be more common than previously appreciated (Chen et al., 2019; Chong et al., 2012; Daneshnia et al., 2023; Alcoceba et al., 2022). Although another explanation for this could be nosocomial transmission of resistant isolates. Evidence has shown that clonal fluconazole-resistant strains can spread between patients and can also be transmitted from contaminated surfaces within hospitals (Hwang et al., 2024; De Carolis et al., 2024). This raises a key question: through what pathways do fungicide-exposed yeast strains transition from the environment to human hosts? The current understanding of the environmental-to-human interface in the emergence of azole cross-resistant opportunistic yeast is outlined in Figure 3.

### 4.1 Food-chain associated transmission of cross-resistant yeast pathogens

One prominent route for the transmission of azole-resistant yeast pathogens to humans is through the food chain. This includes consumption of fruits contaminated with fungicide-resistant yeasts, as has been previously described. Another critical pathway involves the transmission of resistant opportunistic yeast through the consumption of animal-derived food products. *Candida* spp. and other yeast pathogens are well-recognized members of the natural gastrointestinal microbiota of animals, similar to humans (Castelo-Branco et al., 2020; Deng et al., 2025). What is particularly striking is the increasing incidence of azole-resistant *Candida* isolates recovered from livestock, despite systemic antifungal therapy rarely being applied in veterinary contexts (Lima et al., 2022; Castelo-Branco et al., 2022). This paradoxical emergence of azole resistance in animal associated yeast species is likely driven by indirect environmental pressures. Constant exposure to agricultural fungicides, especially DMIs used extensively on crops and pastures, may select for resistant fungal populations within the animal microbiota or the environment they inhabit (Castelo-Branco et al., 2022; Fisher et al., 2018). Additionally, direct transmission from fungicide-exposed environmental yeast populations to animals may occur.

Notably, yeast species such as *C. albicans, P. kudriavzevii, N. glabratus* and *C. parapsilosis* have been implicated in mycotic mastitis in dairy cattle, causing mammary gland infections that reduce milk yield (Devanathan et al., 2024). Resistance profiles of these isolates are concerning: Du et al. (2018) demonstrated that *P. kudriavzevii* isolates from mastitis milk samples were resistant to fluconazole, ketoconazole, and itraconazole, despite the absence of prior antifungal treatment in the affected cows (Du et al., 2018). This suggests environmental stressors, likely prolonged exposure to agricultural DMIs driving azole resistance independently of clinical antifungal use.

Further evidence of environmentally mediated azole resistance is reported in other domestic livestock including pigs, goats, sheep, horses and chickens (Lima et al., 2022; Mba and Nweze, 2019). Mba and Nweze (2019) isolated yeast species (*C. albicans, C. parapsilosis,* and *N. glabratus*) from blood and urine samples of apparently healthy animals in an abattoir setting. Alarmingly, 12 out of 14 isolates from blood showed resistance to multiple azoles including fluconazole, voriconazole, ketoconazole, and itraconazole. These findings raise significant public health concerns because these azole-resistant strains were recovered from animals with no history of infection or antifungal treatment and were considered fit for human consumption (Mba and Nweze, 2019). The circulation of these resistant strains may be further amplified by dissemination via animal waste, environmental contamination, and direct contact between animals and humans, potentially facilitating the spread of resistant fungi along the food chain and environment (Fisher et al., 2022; Verweij et al., 2009).

### 4.2 Environmental modes of transmission of cross-resistant yeast pathogens

The presence of pathogenic yeast in wastewater environments may contribute significantly to the emergence and evolution of azole cross-resistance. This is largely due to the co-occurrence of clinical and agricultural azole compounds in wastewater, which exerts selective pressure on fungal populations and facilitates the enrichment of resistant strains (Assress et al., 2021). Wastewater treatment plants (WWTPs) are largely ineffective at removing many of these compounds; for example, concentrations of fluconazole, propiconazole, and tebuconazole remain largely unaltered after treatment and are subsequently discharged into the aquatic environment (Kahle et al., 2008). The persistence of fungal pathogens in treated effluent has been documented in various regions, including a South African WWTP, underscoring the limitations of current treatment protocols in eliminating fungal pathogens (Assress et al., 2019). Furthermore, during wastewater processing, aerosolization of fungal pathogens, including yeasts, can lead to their dissemination as bioaerosols and dust particulates, posing potential inhalational risks for humans and contributing to the spread of cross-resistant strains (Szulc et al., 2021; Tian et al., 2020).

Agricultural effluent is another major route through which DMIs enter natural water bodies, where they persist and exert non-specific toxic effects (Zhu et al., 2023; Wightwick et al., 2012). Fungicides can enter aquatic systems through surface runoff, and drainage primarily from heavy agricultural areas, or from wastewater treatment discharge (Zubrod et al., 2019). This environmental contamination not only disrupts aquatic ecosystems (Zhu et al., 2023), but also imposes selective pressure on environmental fungal populations, potentially driving the development of azole resistance. River systems polluted with both fungicides and yeast pathogens may therefore serve as important reservoirs and evolutionary hotspots for the emergence of cross-resistant strains.

Soil is also a critical environmental niche for opportunistic yeast, especially given the high persistence and low degradation rates of agricultural fungicides, which accumulate over time and maintain selective pressure (Castelo-Branco et al., 2022; Ockleford et al., 2018). In a 2012 study by Yang et al., *C. tropicalis* strains isolated from agricultural soils in Taiwan exhibited reduced susceptibility to fluconazole in 17 out of 18 samples, and several of these strains were also resistant to the DMIs penconazole and tebuconazole (Mackenzie and Jeggo, 2019). Notably, genotyping these strains revealed close genetic relationships between soil *C. tropicalis* isolates and those from clinical and food sources, such as fruit (Yang et al., 2012; Chen et al., 2019) suggesting potential for environmental-to-human transmission. Similarly, a study from Brazil isolated *C. albicans*, *C. parapsilosis sensu stricto*, *C. tropicalis*, and *C. metapsilosis* from fungicide-treated agricultural soils. All 24 isolates showed resistance to the DMIs tetraconazole and tebuconazole, and several *C. albicans* strains also demonstrated elevated resistance to fluconazole and voriconazole (Sidrim et al., 2021). These findings are concerning within the One Health framework, as they highlight the potential for environmental reservoirs, particularly soil, to harbour medically relevant yeast pathogens with reduced azole susceptibility (Fisher et al., 2022; Yang et al., 2012).

Importantly, these data implicate environmentally derived yeast pathogens as capable of direct transmission to humans via inhalation, ingestion, or contact with open wounds, increasing the risk of human infection with environmentally acquired cross resistance isolates.

### 4.3 Cross-resistant yeast species in the clinic

A 2007 study by Muller et al., investigated azole cross-resistance in human and environmentally sourced yeast species. Specifically, *C. albicans* isolates from HIV-positive patients (+/− treatment) were compared to *C. albicans* from animals, *N. glabratus* from beets, *P. kudriavzevii* from grapes, draff, and grass silage, *C. rugosa* from animal feed, *C. lambica, C. norvegensis*, *C. stellata* from grapes (Muller et al., 2007). As assumed, environmental isolates showed less sensitivity to DMIs, (fluquinazole, penconazole, tebuconazole, and triadimenol). Whereas human isolates were more resistant towards clinical azoles (fluconazole, itraconazole and voriconazole). However, cross resistance was reported for 16/54 environmental isolates (~30%) including all *C. stellata* and *C. lambica* isolates (*n* = 10). Within the clinical group, nearly all isolates from fluconazole-treated patients were resistant to both clinical and agricultural azoles, indicating cross-resistance in the clinic. By contrast, *C. albicans* from animals remained uniformly drug-sensitive (Muller et al., 2007). While only one study has formally reported cross-resistant yeast strains in clinical settings, this likely stems from limited surveillance of clinical isolates. Notably, we have identified multiple CR strains from regional hospitals in Northern Ireland (unpublished data).

### 4.4 Emerging drivers of cross-resistance

Human disruption of the environment is increasingly linked to the emergence of new fungal pathogens. *Candida palmioleophila*, first isolated from soil in 1988, has since been identified as a rare but emerging human pathogen. In 2018, a fluconazole-resistant clinical isolate was reported in China, followed by a 2019 Polish study identifying echinocandin resistance (Wu et al., 2023; Mroczynska and Brillowska-Dabrowska, 2019). This species has been recovered from diverse environmental reservoirs including: soil, marine systems, wastewater, crops, and animals, and displays notable adaptability to harsh conditions (Wu et al., 2023). Wu et al. (2023) suggest that continued anthropogenic pressures, combined with global climate change, may further disturb the ecological niches of *C. palmioleophila*, increasing its potential to emerge as a more significant human pathogen.

It is now recognised that climate change may be contributing to the emergence of thermotolerant fungal pathogens (Casadevall et al., 2019; George et al., 2025; Jackson et al., 2019; Mora et al., 2022). *Candidozyma auris* exemplifies this trend, with its rapid, near-simultaneous emergence on multiple continents and high thermotolerance consistent with environmental selection in a warming world (Casadevall et al., 2019; Jackson et al., 2019). Increasing temperatures, salinity, and pollution are altering fungal community dynamics, enabling opportunistic yeasts such as *Candida*, *Cryptococcus*, and *Rhodotorula* to expand into new ecological and host niches (Casadevall et al., 2019; Mora et al., 2022). Moreover, stressors such as heat and fungicide exposure may accelerate mutagenesis and resistance development (Metcalf et al., 2025). These findings reinforce the need for a One Health framework, as environmental reservoirs of antifungal-resistant yeasts pose an increasing threat to human health (George et al., 2025; Daneshnia et al., 2022).

## 5 Conclusion

The emergence of azole cross-resistant yeast pathogens represents a critical One Health challenge, with wide-reaching implications for human and animal health, environmental integrity, and global food security. This review synthesises current evidence implicating the widespread use of demethylation inhibitor (DMI) fungicides in agriculture as a selective pressure contributing to the evolution of resistance to clinical azoles in yeast species. Structural similarities between agricultural DMIs and medical azoles enable shared resistance mechanisms to be co-selected across environmental and clinical settings. Moreover, environmental cycling of resistant strains, mediated by human–animal–ecosystem interactions, amplifies the risk of resistance dissemination.

Ultimately, despite growing research, the transmission routes linking environmental reservoirs to clinical settings remain poorly understood. Proposed pathways often rely on stochastic exposure and fail to account for the high prevalence of resistant strains in healthcare environments. Notably, wastewater treatment systems require closer scrutiny, as clinically relevant resistant fungi have been detected in treated drinking water (Mhlongo et al., 2019), suggesting that current treatment processes may be insufficient. Robust surveillance across environmental reservoirs, especially wastewater systems, is essential to clarify transmission dynamics.

A central issue is that resistance arising in the environment, beyond direct clinical settings, fundamentally undermines traditional antimicrobial stewardship approaches. Rational prescribing alone cannot contain resistance acquired through fungicide exposure in soil, water, and agricultural run-off. Therefore, a broader strategy is required, one that incorporates comprehensive environmental surveillance, targeted infection prevention, and mitigation of ecological reservoirs of resistance.

The continued use of DMIs to control plant fungal pathogens exacerbates this problem by exerting non-specific pressure on fungal communities. These agents disrupt beneficial saprophytic fungi that help suppress opportunistic pathogens, such as *Candida*, altering fungal ecology and opening niches for resistant organisms to thrive (reviewed in Azevedo et al., 2015; Hof, 2001). Additionally, some DMIs like clotrimazole are known to interfere with mammalian cytochrome P450 enzymes, potentially causing endocrine disruption even at low environmental concentrations (Jørgensen and Heick, 2021; Draskau and Svingen, 2022). Thus, the ecological and toxicological consequences of azole use extend beyond resistance alone.

Reducing reliance on azole fungicides that overlap structurally with clinical antifungals is an obvious but partial solution. Encouraging the use of alternative DMIs or non-azole fungicides could help preserve the efficacy of key medical treatments. However, this strategy fails to fully address the broader environmental impacts of fungicide use and does not resolve the competing imperative of ensuring global food security. With fungal crop pathogens responsible for devastating yield losses worldwide, sustainable agricultural productivity remains non-negotiable.

Many aspects of the One Health framework for cross-resistance in pathogenic yeast remain underexplored. The molecular mechanisms underlying cross resistance are not well defined, it is unclear whether cross resistance constitutes a distinct process, whether specific DMIs differentially select for cross resistance, or if fitness trade-offs shape its evolution. Empirical studies are needed to map the developmental trajectories of cross resistance and assess the stability and transmissibility of cross resistance phenotypes. Additionally, environmental isolates of pathogenic yeast are often dismissed as contaminants, potentially overlooking true ecological niches that may serve as resistance hotspots. Current cross resistance research is largely limited to *N. glabratus* and *C. parapsilosis*, despite global variation in yeast species distribution. Climate differences and patterns of agricultural azole use may further influence cross resistance emergence. Broader surveillance across species, climates, and regions is essential to assess the global risk of cross-resistance.

In conclusion, addressing the threat of cross-resistance requires a multifaceted and integrated approach. Solutions must account for both clinical and agricultural priorities, balancing the urgent need to safeguard antifungal efficacy with the equally pressing demand to secure the global food supply. This will require interdisciplinary collaboration, policy innovation, and investment in sustainable disease control technologies underpinned by a unified One Health framework.
